# Punitive laws, key population size estimates, and *Global AIDS Response Progress Reports*: an ecological study of 154 countries

**DOI:** 10.7448/IAS.20.1.21386

**Published:** 2017-03-17

**Authors:** Sara LM Davis, William C Goedel, John Emerson, Brooke Skartvedt Guven

**Affiliations:** ^a^ Center for Human Rights and Global Justice, New York University School of Law, New York, NY, USA; ^b^ College of Global Public Health, New York University, New York, NY, USA

**Keywords:** Human rights, HIV, key populations, men who have sex with men, law, indicators

## Abstract

**Introduction**: UN global plans on HIV/AIDS have committed to reducing the number of countries with punitive laws criminalizing key populations. This study explores whether punitive laws are associated with countries’ performance on targets set in the global plans.

**Methods**: The study used chi-square tests of independence to explore associations between legal status, key population size estimates, and HIV service coverage for 193 countries from 2007 to 2014. We used data reported by countries on United Nations *Global AIDS Progress Report* **(GARPR)** indicators, and legal data from UNAIDS, UNDP, and civil society organizations. Due to lack of sufficiently reliable legal data, only men who have sex with men (MSM) could be studied. The study utilized public data aggregated at the national level. Correspondence with individual experts in a subset of countries stated the purpose of the study, and all responses were anonymized.

**Results and Discussion**: A significantly larger proportion of countries that criminalize same-sex sexual behaviour reported implausibly low size estimates or no size estimates for MSM. This is consistent with findings in qualitative research that MSMs are marginalized and reluctant to be studied in countries where same-sex sexuality is criminalized. Size estimates are often used as the denominators for national HIV service coverage reports. Initially, countries that criminalized same-sex sexuality appeared to have higher HIV testing coverage among MSM than did countries where it is not criminalized. However, investigation of a subset of countries that have reported 90–100% HIV testing coverage among MSM found that most were based on implausibly low or absent size estimates.

**Conclusions**: Criminalization of same-sex sexuality is associated with implausibly low or absent MSM size estimates. Low size estimates may contribute to official denial of the existence of MSM; to failure to adequately address their needs; and to inflated HIV service coverage reports that paint a false picture of success. To enable and measure progress in the HIV response, UN agencies should lead a collaborative process to systematically, independently and rigorously gather data on laws and their enforcement.

## Introduction

In endorsing universal access to healthcare as a Sustainable Development Goal (SDG), United Nations (UN) member states resolved to “leave no one behind”[1]. To achieve this in the global HIV response, the Joint UN Programme on HIV and AIDS (UNAIDS) 2016–21 strategy urges countries to focus on reaching key populations: men who have sex with men (MSM), sex workers, people who inject drugs, and transgender people [2,3]. A target in the strategy aims to ensure that “90% of key populations … have access to HIV combination prevention services” [4].

However, extensive research by UN agencies, civil society and academic research has found that punitive laws criminalizing behaviour of key populations impede their access to HIV services. The UN Special Rapporteur on the Right to Health and the Global Commission on HIV and the Law, as well as numerous civil society organizations, have compiled exhaustive studies showing that punitive laws, and the abuses that accompany their enforcement, lead key populations to remain underground and to avoid government-run HIV prevention, treatment and care programmes [5–7]. Similarly, in their 2016 update to the 2012 *Lancet* Series on gay, bisexual and other MSM, Beyrer and colleagues report that MSM “have disproportionately high burdens of HIV infection”, linking this in part to a “marked increase in anti-gay legislation in many countries” [8]. The World Health Organization (WHO) has called for reform of punitive laws in order to improve access to HIV services by all key populations [9].

Similar reforms are also urged in the UNAIDS 2016–21 strategy, and the UNAIDS 2011–15 strategy included a target to reduce the number of countries with punitive laws affecting key populations by half [3,10]. UNAIDS recommends including “seven key programmes” to reduce stigma and discrimination [11]. The Global Fund to Fight AIDS, TB and Malaria has committed to promoting and protecting human rights and gender equality in its 2017–22 strategy, with an institutional Key Performance Indicator to monitor countries’ investment in the UNAIDS seven key programmes [12].

Despite these high-level commitments, research to quantify the specific impact of laws on HIV remains in the early stages of development. Recent work has begun to explore this area [13–15]. In particular, Schwartz and colleagues found that criminalization of homosexuality in Nigeria negatively impacted on HIV service uptake by MSM [16]. However, some researchers continue to question whether the impact can be measured at all [17]. UNAIDS and WHO do not yet routinely integrate analysis of laws into the data and assumptions underlying the modelling of the HIV epidemic, in part because the specific impact of laws on the epidemic has been difficult to quantify [18].

This article aims to advance the analysis of the relationship between punitive laws and performance of national HIV programmes by exploring potential associations between national laws and national performance on HIV targets. The law represents only one part of a complex legal environment that includes law enforcement, judicial interpretation of the laws, access to legal representation, and more. However, analysis of the impact of the law itself is a first step in analyzing this more complex environment. Demonstrating that the impact of law on HIV can be quantified should also help to make the case for law reform that WHO, UNAIDS, and many others have urged based on qualitative evidence.

### Methods

The 2011–15 Political Declaration on HIV and AIDS, the UNAIDS 2011–15 strategy, and the more recent 2016–21 Political Declarations and UNAIDS strategy have all included targets for reform of punitive laws affecting key population. This ecological study aimed to use statistical testing to assess differences in HIV data reported against indicators across categories of legal statutes for all four key population groups.

The advantage of this methodology is that it highlights data gaps and patterns at the global scale. A disadvantage is that it does not provide insight into HIV vulnerability at the individual level. In this case, the methodology highlighted lack of sufficient HIV and legal data for most key population groups. This meant that the study was only able to conduct statistical testing for MSM. The data and gaps identified are discussed in more detail below.

### HIV data on key populations

We obtained HIV data reported by countries to UNAIDS for all four key population groups: men who have sex with men, sex workers, people who inject drugs, and transgender people.

UNAIDS sets global HIV indicators in *Global AIDS Response Progress Report* (GARPR) guidelines [19]. In the current strategy, these indicators measure, among other things, progress made by countries towards the so-called “90–90–90” targets: 90% of people living with HIV tested, 90% of those who test positive on antiretroviral treatment, and 90% of those on treatment showing reduced viral loads. In the 2016–21 strategy, an additional Target 6 aims to ensure that 90% of key populations have access to combination prevention services [4]. These targets are based on modelling that shows that through rapid scale-up of coverage, “The AIDS epidemic can be ended as a public health threat in all places and among all populations by 2030”. [4] National targets are informed by global targets and by WHO target-setting guidelines [20].

Member states submit the GARPR using a combination of narrative country progress reports and quantitative tools, including the Spectrum computer package. Initially, countries reported every second year. Since 2013, countries have reported annually. In 2004, only 102 countries reported data; by 2014, 180 countries did. The full database is published by UNAIDS online through AIDSInfo [21]. UNAIDS also publishes narrative country progress reports on its website.

We used the database on AIDSInfo, focusing on the data reported for sex workers, men who have sex with men, transgender people, and people who inject drugs. We looked at the country-reported data for four GARPR indicators: size estimates, condom use coverage, HIV testing coverage, and HIV prevalence.

Not all countries report on all indicators, and there were numerous gaps on data for key populations across the board. Overall, more countries had reported on MSM than on sex workers and people who inject drugs. At the time of the study, there was no published data on transgender people on AIDSInfo. UNAIDS shared unpublished data on transgender people, but only twenty countries had ever reported transgender size estimates, and only six of these had also reported on HIV prevalence among transgender people. All these reports were only for one year, 2015.

Where both HIV and legal data on key populations were available for multiple years, we used the most recent reports for the same year. Where legal data was available for only one year, we used the most recent HIV data relevant to that year.

To assess reported size estimates as a proportion of total national populations, we used country reports to the UN Statistical Division [22]. The UN Statistical Division provides official population estimates from 1950 to 2010 and projections for all years thereafter. We matched these estimates and projections to size estimates based on reporting year. Size estimates for MSM were examined as a proportion of the male population. However, it is worth noting that UN Statistical Division data were not disaggregated by age, and MSM size estimates are often for the 15–49 age range. Size estimates were considered plausible for this study when they represented more than 1.0% of the male population. (Published guidance by UNAIDS and WHO does not establish a threshold for plausibility of size estimates. The threshold of 1% used in this study was established based on UNAIDS unpublished data.)

### Legal data

The review of legal data highlighted significant gaps in the strategic information needed to monitor UN commitments in the HIV response. As noted, the 2011 UN *Political Declaration on HIV and AIDS* committed to monitoring the impact of the legal environment on HIV prevention, treatment, care and support; based on this, the UNAIDS 2011–15 strategy committed to reducing by half the number of countries with punitive laws [23]. However, our review found that no UN agency had systematically mapped which countries have these punitive laws. While civil society organizations have conducted some mapping, this has not been systematically done for all key populations and for all countries, and it has rarely been done in a sustained way for more than one year. As a result, data was insufficient to conduct statistical testing for any key population group other than MSM. This section discusses some of the sources reviewed and the challenges with each.

Our review began with the UN human rights treaty bodies. The UN Committee on Economic, Social and Cultural Rights is the treaty body that monitors States Parties’ implementation of the *International Covenant on Economic, Social and Cultural Rights*, which upholds the right to highest attainable standard of health [24]. This and other treaty bodies, including the Human Rights Committee, Committee on the Elimination of Discrimination Against Women and Girls, and others, routinely monitor and report on countries’ human rights records. As part of their review process, the treaty bodies also review submissions from civil society, and independently review and verify country reports on their progress. While the process is rigorous and independent, it produces insufficient data for this study. This is due to the fact that states are reviewed only once every four years, and the Committee monitors many economic and social rights in addition to the right to health. While some recommendations from the treaty bodies reference HIV and human rights, the recommendations tend to be general in nature, and do not produce the kind of comprehensive legal data set that would permit statistical testing.

The UN Office of the High Commissioner for Human Rights (OHCHR) also does not engage in routine monitoring or analysis of laws impacting HIV. In fact, OHCHR terminated its sole staff position on HIV and human rights in 2013. OHCHR’s Born Free and Equal program, which promotes LGBT rights, has published a map showing criminalization of same-sex sexual behaviour from 1799 to the present [25]. However, our review of the source data found much of it derived from published reports by the International Lesbian, Gay, Bisexual, Trans and Intersex Association (ILGA) [26]. Additional data came not from the laws themselves, but from unreliable secondary and tertiary sources, such as Wikipedia. This did not produce a reliable data set.

We then examined research produced by UNAIDS and the co-sponsors of the Joint UN Programme on HIV and AIDS, which brings together 11 UN organizations; UNDP’s scope of work within the Joint UN Programme includes addressing human rights and gender equality in the HIV response. UNAIDS has itself published extensive research and guidance notes on law and HIV, especially on the issue of HIV-related travel restrictions [27]. However, we found that while both UNAIDS and UNDP have conducted and documented extensive advocacy on the law in HIV, neither agency has conducted systematic, regular monitoring of the punitive laws that affect key populations, leaving a gap in the strategic information needed to monitor implementation of the commitments made in the Political Declaration and the UNAIDS Strategy.

In fact, UNAIDS did not set a baseline for the 2011–15 target of “reducing by half the number of countries with punitive laws”, or routinely and systematically report on progress towards the target [28].

UNAIDS did require, as part of the GARPR system, that countries report on laws pertaining to the HIV response using the National Commitments and Policies Index (NCPIs), a narrative questionnaire. The NCPIs are completed by government officials, who may opt to include civil society in the reporting process if they wish. Not all countries include civil society input in all reports.

Moreover, in contrast with the independent review process used by UN human rights treaty bodies, the legal data reported by countries on the NCPIs are not independently reviewed or validated by UN human rights experts. This lack of independent review is reflected in the unreliable quality of the legal data in the NCPI reports. We found inconsistencies between countries’ characterization of their own laws, and more critical analyses of the same countries’ laws by other independent sources, including credible nongovernmental organizations. For example, China’s most recent NCPI states that China has harm reduction policies, without explicitly acknowledging that thousands of Chinese people who inject drugs are also detained in abusive compulsory drug detention centres [29]. Uganda’s 2013 NCPI stated that there were no “laws, regulations or policies that present obstacles to effective HIV prevention, treatment, care and support” for men who have sex with men, though in fact same-sex sexuality has been criminalized in Uganda since 1950 [30].

We also found that some NCPI questions were phrased too generally to produce meaningful legal data for the purposes of this study. For example, the NCPI categorizes criminalization of sex work as a “yes/no” question, but laws on sex work frequently involve a diverse range of acts, actors and venues. By contrast, the Sex Work Law Map, discussed further below, uses 11 categories of criminalization for its mapping [31]. A UNAIDS poster, “Making the law work for the HIV response”, drew on NCPI reports to chart categories of protective and punitive laws by country [32]. However, only one year of data (2010) was available, insufficient for statistical analysis.

The Global Commission on HIV and the Law produced a comprehensive overview of the global state of HIV and the law in 2012, which called for reform of punitive laws that affect key populations [33]. United Nations Development Programme (UNDP) has engaged in ongoing work with countries to promote law reform. However, the Commission does not produce annual mapping of punitive laws affecting key populations of the kind required for this study. A UNDP-led process, the Legal Environment Assessment, is used to assess national laws affecting HIV and to mobilize national political will towards the reform of laws that hinder the response [34]. However, the process produces narrative reports which are more broadly descriptive, and which like the NCPIs are also not independently verified by human rights experts. While these are also valuable advocacy tools, they do not constitute systematic annual mapping of laws of the kind required for statistical analysis.

As there were no UN data sets on laws affecting key populations, we reviewed civil society sources. These included the above-mentioned work by ILGA; the Sex Work Law Map; and mapping of penalties for drug use in the European Union and African Union conducted by the European Monitoring Centre for Drugs and Drug Addiction and by the International Council of AIDS Service Organizations, reporting by the Global Network of People Living with HIV (GNP+), as well as reporting by other civil society networks, including some supported by UNAIDS [35–40]. However, while these reports were overall systematic and independent in approach, their definitions were not consistent; in most cases, each network had been resourced to map only a limited number of countries for a limited number of years (in some cases, only one year). Thus, most offered insufficient legal data to enable statistical testing.

Based on this comprehensive review of UN and civil society sources, we found that only the International Lesbian, Gay, Bisexual, Trans and Intersex Association (ILGA) had created an independent, systematic and useable data set on laws that criminalize behaviour of a key population group, MSM. ILGA has also published a report on these laws annually for many years, creating a significant body of legal data. Arguably, ILGA’s task was simpler than that facing other groups, as legal provisions that criminalize same-sex sexuality tend to be briefer, less complex to analyze, and less varied than are laws criminalizing sex work and drug use.

As noted above, an advantage of the ecological study method is that it identifies gaps in data. In this case, it identified gaps in HIV data, especially for transgender people; we found that more countries reported on HIV data for MSM in the GARPR reports than other key population. It also identified gaps in legal data for all key populations: While there has been significant qualitative research into human rights issues and advocacy around them related to the HIV response, no UN agency has conducted systematic, routine mapping of punitive laws. Civil society has made an effort to fill this gap, but these efforts have been piecemeal, except in the case of MSM. Thus we used legal data from 2007 to 2014 published by ILGA. 154 of the countries mapped by ILGA had also reported HIV data for MSM in their GARPR reports, creating a sufficient quantity of legal and HIV data to allow statistical testing.

### Statistical analysis

Data were extracted into Microsoft Excel and transformed into categorical variables. For example, reported estimates for HIV testing coverage were transformed from their raw values into four groups (estimates less than 25%; estimates between 25.0% and 49.9%; estimates between 50.0% and 74.9%; Estimates greater than or equal to 75.0%). As both health and legal variables are categorical, chi-square tests of independence were used to assess associations between GARPR indicators and legal statutes. Analyses were conducted in IBM SPSS 23.0 (IBM Corp., Armonk, NY).

### Ethics and consent

The study utilized public data aggregated at the national level. Correspondence with individual experts stated the purpose of the study, and all responses were anonymized.

## Results and discussion

UNAIDS’ online portal for aggregation of HIV data, AIDSInfo, included four GARPR indicators specific to MSM: population size estimates, HIV testing coverage, HIV prevalence, and condom use. The methodology used in this study permits the mapping of gaps and patterns in global data, but does not provide insight into individual vulnerability to HIV. No significant associations were observed between the legal status of same-sex sexual behaviour and reported estimates for HIV prevalence and condom use among MSM. We did find associations between punitive laws, size estimates and HIV testing coverage of MSM, which are discussed below.

### Key population size estimates

Criminalization of same-sex sexuality is associated with implausibly low reported size estimates for MSM as less than 1% of the overall population. A larger proportion of countries with laws criminalizing same-sex sexuality reported implausibly low MSM size estimates than did countries without such laws, χ 2 (2) = 16.182, p **<** .001 (see Table 1).

**Table 1.** Reported size estimates for men who have sex with men (MSM) grouped according to legal status of same-sex sexuality in the country (2007–14)

MSM size estimates as proportion of male population:	Countries where same-sex sexual behaviour was legal	Countries where same-sex sexual behaviour was criminalized, punished with imprisonment/fines	Countries where same-sex sexual behaviour was criminalized, punished with death penalty
Less than 1.0%	50.0%	87.1%	100.0%
1.0% or higher	50.0%	12.9%	0.0%

χ^2^(2) = 16.182, *p* < .001.

**Figure 1.**
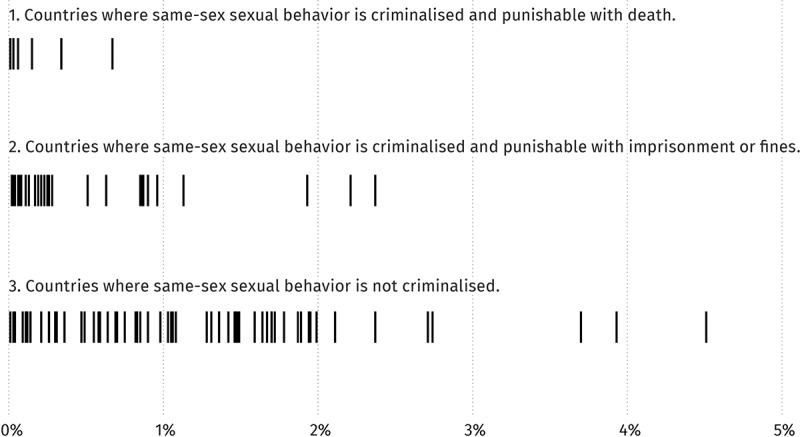
**Relation between criminalization of same-sex sexuality and country-reported population size estimates for men who have sex with men (2007–14)**.

Specifically, 100% of countries that punish same-sex sexual behaviour with the death penalty reported implausibly low or had no MSM size estimates (Figure 1, line 1). 87.1% of countries that punish same-sex sexual behaviour with imprisonment or fines reported implausibly low or no size estimates for MSM (Figure 1, line 2). Only 50.0% of countries where same-sex sexual behaviour was not criminalized reported implausibly low or no size estimates (Figure 1, line 3). Thus, the threat of the death penalty is also associated with lower MSM size estimates.

These findings are consistent with qualitative research that has found that MSM are hidden and reluctant to be studied in countries where they face risk of arrest. In cases where countries published no MSM size estimates, it is possible that this was because the risk to such populations made conducting studies inadvisable. Other countries may have conducted size estimates but not published the results, due to concerns about the public backlash the results could generate.

Key population size estimates are critically important information for evaluation of progress in the HIV response, and are often the basis for national planning and resource allocation for targeted programmes. These size estimates also form the denominator for service coverage reports, including coverage of HIV testing and condom use.

### HIV testing coverage

Success in implementing the “Fast Track Approach” is dependent in part on “a global effort to close gaps in the treatment cascade”, in particular through increased HIV testing of key populations to enable early access to antiretroviral treatment [4]. As noted above, 90% HIV testing coverage is the first target in the UNAIDS 90-90-90 targets. Failure to diagnose HIV status naturally affects the rest of the cascade, and makes it difficult for countries to reach or measure the other two targets of 90% treatment coverage and 90% viral load reduction.

**Figure 2.**
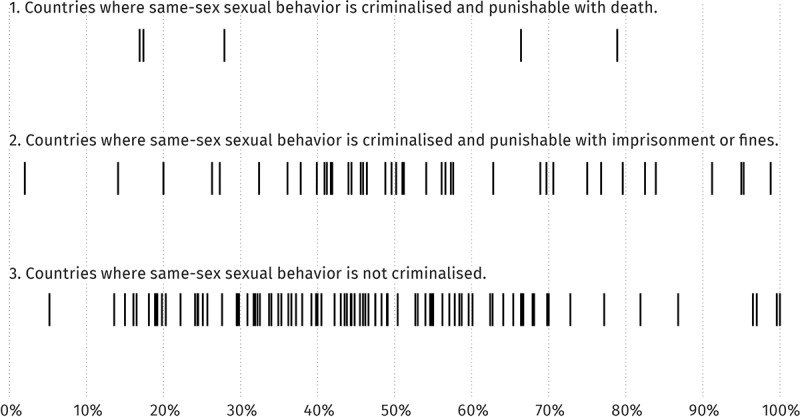
**Relation between criminalization of same-sex sexuality and country-reported HIV testing coverage of men who have sex with men (MSM) (2007**–**14)**.

Initially, countries’ reported rates of HIV testing coverage among MSM also appeared to vary slightly with the legal status of same-sex sexual behaviour (Table 2, Figure 2). Statistical testing found that a larger proportion of countries that criminalize same-sex sexual behaviour (17.8%) reported very high HIV testing coverage among MSM. Countries that did not criminalize same-sex sexuality reported lower HIV testing coverage for MSM (6.5%), χ^2^(6) = 15.904, *p* = .014. (See Figure 2, line 2)

**Table 2.** Relation between laws criminalizing same-sex sexuality and country-reported HIV testing coverage of men who have sex with men (2007–14)

Country-reported HIV testing coverage among MSM	Countries where same-sex sexuality was legal	Countries where same-sex sexual behaviour was criminalized, punished with imprisonment/fines	Countries where same-sex sexual behaviour was criminalized, punished with death penalty
Less than 25·0% reported HIV testing coverage among MSM	16·3%	13·3%	66·7%
25·0–49·9% reported HIV testing coverage among MSM	54·3%	54·2%	16·7%
50·0–79·9% reported HIV testing coverage among MSM	22·8%	26·7%	16·7%
80·0% or greater reported HIV testing coverage among MSM	6·5%	17·8%	

χ^2^(6) = 15·904, *p* = ·014.

These findings were not consistent with findings from qualitative research, and differed from the above-mentioned findings on condom use and HIV prevalence among MSM, which showed no difference across categories of criminalization.

To understand the results, we probed the data further, focusing on the high end of the spectrum which distorted the overall distribution of data: the twelve countries that reported 90% or higher HIV testing coverage among MSM during 2007–14 (Figure 2, lines 2 and 3, right-hand side of the chart). The countries were Algeria, Angola, Antigua and Barbuda, Bosnia and Herzegovina, Indonesia, Mauritius, Panama, Saint Kitts and Nevis, and Suriname. Three countries had also reported 100% coverage: Hungary, Saint Lucia and Marshall Islands. Seven of these twelve countries criminalize same-sex sexuality, and five do not.

Seeking clarification, we wrote by electronic mail to domestic AIDS experts, including UNAIDS country and regional directors. We also wrote to civil society organizations which represent or work closely with MSM on the HIV response in the 12 countries. We requested their explanation as to why the countries had reported such high HIV testing coverage among MSM.

Several UNAIDS country directors and civil society organizations responded that in their opinions, the high HIV testing coverage reported was inaccurate and was not supported by data. A Hungarian and an Algerian civil society organization each shared independently published studies with findings of significantly lower HIV testing coverage for MSM than those reported by Hungary and Algeria  to UNAIDS [38,39].

Several of the civil society respondents commented that in their experience, the punitive laws in their countries made conducting accurate HIV size estimates for MSM challenging, and noted as factors the lack of funding for community-led work with MSM, the lack of privacy in close-knit societies, and the perception that criminalization publicly legitimizes high degrees of stigma and discrimination against MSM.

The high reported coverage appeared linked to low or absent denominators in epidemiological studies, including MSM size estimates. Seven of the twelve countries reporting 90–100% HIV testing coverage had no MSM size estimate at the time of their high coverage report. We reviewed GARPR narrative reports for the relevant reporting years, and found no reports of special efforts to reach MSM in the 12 countries. Some, such as the GARPR for the Marshall Islands, lacked any size estimate, HIV prevalence or condom use data for MSM, suggesting that the high reported HIV testing coverage of MSM was not supported.

Some of the twelve countries had used single surveys with small denominators as a basis for national coverage reports to UNAIDS. For example, Hungary’s 2010 narrative GARPR report states that their 100% reported HIV testing coverage of MSM for that year was based on a single three-month survey of 388 “homo/bisexuals” [40]. Algeria conducted one survey of 59 men, of whom 57 retrieved their test results, resulting in a 96.6% national coverage estimate [41]. Indonesia based their reported 2011 national coverage rate of 92% on an Integrated Bio-Behavioral Survey, which found that 39% of MSM had taken an HIV test, 92.96% of whom had retrieved their results. The percentage of respondents who had retrieved their tests appeared to have been mistakenly reported by Indonesia as the national HIV testing coverage [42].

In sum, we found that criminalization of same-sex sexuality is associated with implausibly low or absent size estimates for MSM; and in turn, that lack of plausible size estimates is a factor in implausibly high HIV testing coverage.

## Conclusions

This study highlights the need for more reliable and rigorously collected HIV and legal data in order to ensure progress towards global objectives set out in the new *Political Declaration on HIV and AIDS*.

First, this study identifies some significant gaps in reliable, nationally reported data for all key populations, not just MSM, and the need for care and scepticism when using this data. Accurate population size estimates are key to national planning and resourcing, and as a denominator for service coverage, they are the basis for measuring progress in fulfilment of health targets for key populations. The fact that only 20 countries had ever reported to UNAIDS on transgender people is symptomatic of a yawning global vacuum in services for a population estimated to have 19% HIV prevalence globally [43]. However, such data is produced in a political context. As Baral and Greenall argue, key populations suffer from a data paradox: “Decision-makers deny that most affected populations exist … so no research gets done on these populations; the lack of data feeds the denial; and so on” [44]. These findings also highlight the need to view reports of extraordinary success with critical eye. Unfortunately, in a context in which countries seek global prestige, ambitious global targets may create perverse incentives, even more if they are monitored through a process that relies on self-reporting.

Second, given the demand for quantification of the impact of human rights on HIV and the ambitious targets set in the new Political Declaration and UNAIDS Strategy, our findings point to the need for a new, higher-level initiative to produce strategic information for the global HIV response that includes systematic, regular and objective monitoring of legal data. UNAIDS, members of the Joint UN Programme, and UNOHCHR should lead a process of systematic mapping of laws relevant to HIV, as well as specific steps countries are taking to reform these laws. This should include laws that criminalize key populations, but also laws that impede other aspects of the HIV response, such as laws on procurement. While the NCPIs attempted to do this, the lack of independent review of country-reported data has made them unreliable.

UN agencies may not be sufficiently resourced to do all such mapping themselves. However, they could lead a network that brings together UN agencies, civil society and academic researchers working in partnership, to define, categorize, and analyze such laws, publishing results online for public access. A potential model for this type of collaboration is Cochrane, which coordinates networks to produce independent, systematic reviews of health research.

A growing body of literature points to the ways in which numerical indicators, increasingly used as tools of global governance, mask political complexities on the ground [45,46]. This study highlights several ways in which politics shape HIV metrics. With the launch of the SDGs and the new Global AIDS strategy, there will be growing pressure on countries to produce global health data to demonstrate progress. In order to be successful, such efforts must take into account the real-world political factors, including laws, that shape both access to HIV services and countries’ reports on their progress.

## Abbreviations

GARPR, Global AIDS Response Progress Report; ILGA, International Gay, Lesbian, Bisexual and Transgender Association; MSM, Men who have sex with men; NCPI, National Commitments and Policies Index; OHCHR, UN Office of the High Commissioner for Human Rights; SDG, Sustainable Development Goals; UNAIDS, Joint UN Programme on HIV and AIDS
